# RAG in clinical practice: a cautionary tale of AI ‘Truthfulness’

**DOI:** 10.1038/s44401-026-00115-x

**Published:** 2026-07-03

**Authors:** HyoJe Jung, Kyusok Cho, Tae Joon Jun, Young-Hak Kim

**Affiliations:** 1 Department of Information Medicine, Asan Medical Center, University of Ulsan College of Medicine, Seoul, Republic of Korea; 2 Research & Business Development Center, Asan Medical Center, Seoul, Republic of Korea; 3 Division of Cardiology, Department of Internal Medicine, Asan Medical Center, University of Ulsan College of Medicine, Seoul, Republic of Korea

**Keywords:** Health care, Mathematics and computing, Medical research

## Abstract

Retrieval-augmented generation (RAG) aims to curb large language models (LLMs) hallucinations, yet its conversational reliability is uncertain. We tested a clinical RAG by executing the same query 100 times at varying dialogue lengths. The hallucination rate surged from 5%(no history) to 40% with just 10 prior exchanges, revealing a critical failure mode. Rigorous conversational testing is essential for patient safety before clinical deployment of RAG systems.

LLMs have the potential to transform clinical medicine^1^, yet their tendency for hallucination presents a substantial barrier to safe adoption^2–4^. RAG has emerged as a key architectural solution^5^, designed to mitigate this risk by grounding model outputs in curated, evidence-based knowledge sources^6–9^. While this approach is an influential tool for clinical decision support^10,11^, its reliability is not absolute. The probabilistic nature of its text generation remains vulnerable to inaccuracies, an issue magnified in conversational settings where accumulating dialogue history can cause contextual drift^12^. To date, existing evaluations of RAG systems have predominantly focused on single-query accuracy, leaving their longitudinal robustness in conversational settings—a critical requirement for patient safety—under-explored. This study directly examines this potential failure mode by testing the hypothesis that accumulating dialogue history degrades performance in a high-precision medication dosing scenario, using the experimental workflow depicted in Fig. 1.

**Fig. 1:**
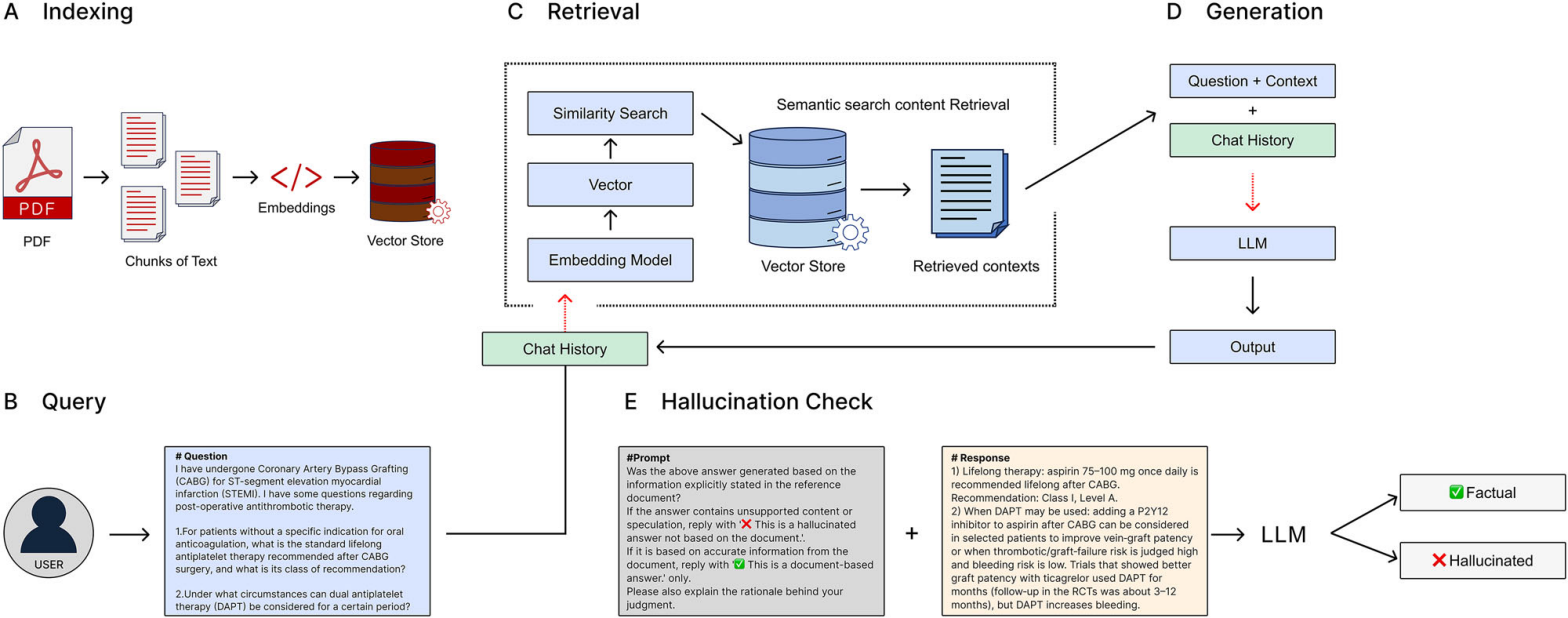
Schematic of the conversational RAG system and experimental workflow. The system architecture consists of (**A**) an indexing pipeline where the source document is chunked, vectorized, and stored; (**B**) a standardised user query; **C** a retrieval step where chat history influences the semantic search for relevant contexts; **D** a generation step where the LLM uses the query, retrieved contexts, and chat history to formulate an output; and **E** an automated evaluation step to check for hallucinations.

Our experiment revealed a dramatic degradation in the RAG system’s factual consistency as the conversational history increased. **For** each tested length of dialogue history, we executed the same standardised query 100 times to assess the stability of the response. At baseline, with no preceding dialogue, the system was highly reliable, exhibiting a hallucination rate of only 5% (i.e., 5 of 100 executions were hallucinatory). However, upon the introduction of just 10 prior question-answer pairs into the context, the system’s performance sharply declined, with the hallucination rate markedly increasing to 40%—an eightfold rise. As the dialogue history expanded further to 50 pairs, the hallucination rate remained significantly elevated, fluctuating between 37% and 44%, demonstrating a consistent state of degraded performance (Fig. 2).

**Fig. 2:**
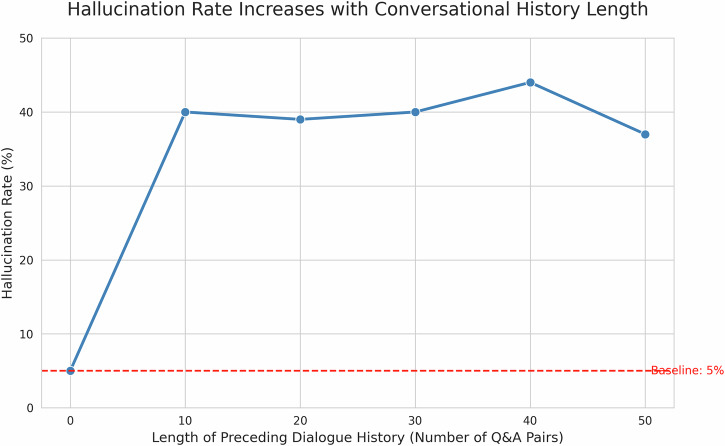
Degradation of factual consistency with increasing conversational history. This line plot illustrates the impact of accumulating dialogue history on the factual accuracy of the conversational RAG system. The *x*-axis represents the number of preceding question-answer pairs. The *y*-axis shows the hallucination rate (%), where each data point represents the percentage of hallucinatory responses from 100 executions of the same query under that specific conversational history length.

A qualitative comparison illustrates the nature of this performance decay. The response generated at baseline was factually correct and directly supported by the retrieved source documents from the ESC guideline. In contrast, the response generated after a preceding history of 10 question-answer pairs contained clinically misleading information, such as an incorrect recommendation class for a treatment (Table 1). Further analysis suggests that this degradation was primarily caused by retriever failure. With an extended conversational history, the retriever’s focus drifted, causing it to retrieve less relevant document chunks that were more semantically related to the noisy context of the preceding dialogue than to the specific query being asked. This retrieval of irrelevant context appeared to be the direct cause for the generation of hallucinated responses (Table 2).

**Table 1:** Qualitative comparison of response degradation.

Length of dialogue history	Dialogue history = 0 (Baseline)	Dialogue history = 10
Query	I have undergone Coronary Artery Bypass Grafting (CABG) for ST-segment elevation myocardial infarction (STEMI). I have some questions regarding post-operative antithrombotic therapy. 1. For patients without a specific indication for oral anticoagulation, what is the standard lifelong antiplatelet therapy recommended after CABG surgery, and what is its class of recommendation? 2. Under what circumstances can dual antiplatelet therapy (DAPT) be considered for a certain period?	
Generated answer	1) Recommendation after CABG (no indication for OAC) - Aspirin 75–100 mg once daily lifelong.\n- Class I, Level A. 2) When DAPT can be considered and for how long - If the CABG was performed in the setting of acute coronary syndrome (e.g. STEMI), standard post-ACS therapy is DAPT (aspirin + oral P2Y12 inhibitor) for 12 months. - Separate from the ACS indication, randomised data show ticagrelor-based DAPT can improve vein-graft patency versus aspirin alone (follow-up in trials 3–12 months) but with increased bleeding. Therefore, short-term DAPT (months) may be considered in selected patients after CABG to reduce graft occlusion or when ischaemic risk is high and bleeding risk is low. - Choice and duration must balance ischaemic benefit against bleeding risk and should be individualised in discussion with your surgical/cardiology team.	1) Lifelong single antiplatelet agent after CABG (no other indication for OAC) - Aspirin 75–100 mg once daily is recommended lifelong. - Strength: Class I, Level A. 2) When short-term DAPT (aspirin + P2Y12 inhibitor) is considered after CABG, and for how long - After an acute coronary syndrome (eg, STEMI), the standard is DAPT for 12 months (aspirin + a P2Y12 inhibitor). This is the routine ACS recommendation (Class I). - Separately, short-term DAPT to improve vein-graft patency or reduce ischaemic events may be considered in selected CABG patients at high ischaemic risk and low bleeding risk (e.g., recent MI/ACS, prior MI, other markers of high recurrent ischaemia). Evidence is inconsistent; this is a conditional recommendation (Class IIb, Level B). Trial follow-up periods and published protocols used durations of roughly 3–12 months (many studies in the 3–12 month range), so clinicians commonly consider DAPT for a few months up to 12 months depending on indication and bleeding risk. - Always balance the potential graft/ischaemic benefit against the increased bleeding risk.
Evaluator’s verdict	**Factual**	**Hallucination**
Evaluator’s rationale	- The recommendation for lifelong aspirin 75–100 mg daily after CABG with Class I, Level A is explicitly stated in the document. - The statement that post-ACS standard therapy is DAPT (aspirin + a P2Y12 inhibitor) for 12 months (with or without PCI) is directly taken from the document. - The discussion that DAPT (particularly ticagrelor-based) can improve vein-graft patency 3–12 months after CABG but increases bleeding (BARC 2–5, not 3–5) and shows no difference in major clinical endpoints is exactly reflected in the cited meta-analysis from the document.	- After CABG, aspirin 75–100 mg daily lifelong (Class I, Level A). - Pre-op discontinuation intervals: prasugrel ≥7 days, clopidogrel ≥5 days, ticagrelor ≥3 days; DOACs 1–2 days depending on renal function. - DAPT after ACS is 12 months; evidence that DAPT vs aspirin alone may improve graft patency after CABG with 3–12 month follow-up and more BARC 2–5 bleeding. - Not explicitly stated in the provided text: - Assigning Class IIb, Level B to the consideration of DAPT after CABG. - Stating ACS DAPT as ‘Class I’ (the excerpt mentions duration but does not give a class/level).

**Table 2:** Analysis of retriever failure due to contextual drift.

Length of dialogue history	Dialogue history = 0 (Baseline)	Dialogue history = 10	
Query	I have undergone Coronary Artery Bypass Grafting (CABG) for ST-segment elevation myocardial infarction (STEMI). I have some questions regarding post-operative antithrombotic therapy. 1. For patients without a specific indication for oral anticoagulation, what is the standard lifelong antiplatelet therapy recommended after CABG surgery, and what is its class of recommendation? 2.Under what circumstances can dual antiplatelet therapy (DAPT) be considered for a certain period?		
Retrieved chunk	monotherapy.\nI\nA\nAfter CABG, aspirin 75–100 mg daily is recommended lifelong.\nI\nA\nIn patients without prior MI or revascularization but with evidence of significant obstructive CAD, aspirin 75–100 mg daily is recommended \nlifelong.\nI\nB\nIn CCS patients with no indication for oral anticoagulation, DAPT consisting of aspirin 75–100 mg and clopidogrel 75 mg daily for up to 6 \nmonths is recommended as the default antithrombotic strategy after PCI-stenting.\nI\nA\nIn patients at high bleeding risk, but not at high ischaemic risk, it is recommended to discontinue DAPT 1–3 months after PCI and to \ncontinue with single antiplatelet therapy.\nI\nA\nIn CCS patients with a long-term indication for OAC, an AF therapeutic dose of VKA alone or, preferably, of DOAC alone (unless \ncontraindicated) is recommended lifelong.\nI\nB\nIn patients with an indication for OAC who undergo PCI, initial low-dose aspirin once daily is recommended (loading dose when not on’)		therapy in patients with AF, history of MI, and at high \nrisk of recurrent ischaemic events who do not have a \nhigh bleeding risk.\nIIb\nB\nAntithrombotic therapy post-percutaneous coronary intervention in chronic coronary syndrome patients and an indication for oral \nanticoagulation—Section 4\nAfter uncomplicated PCI, early cessation (≤1 week) of \naspirin and continuation of dual therapy with an OAC \nand clopidogrel should be considered if the risk of stent \nthrombosis is low, or if concerns about bleeding risk \nprevail over concerns about the risk of stent \nthrombosis, irrespective of the type of stent used.\nIIa\nB\nAfter uncomplicated PCI in CCS patients with \nconcomitant indication for OAC: \n• early cessation of aspirin (≤1 week);\n• followed by continuation of OAC and clopidogrel: \n◦up to 6 months in patients not at high ischaemic \nrisk or\n◦up to 12 months in patients at high ischaemic risk;\n• followed by OAC alone; is recommended
Rationale	The answer to the first question can be found. 1. Source Content: After CABG, aspirin 75–100 mg daily is recommended lifelong. I A 2. Answer: After CABG surgery, aspirin 75–100 mg daily is recommended lifelong. (Class of Recommendation: Class I, Level of Evidence: A)		1. Different Medical Procedure: Your questions are about antithrombotic therapy after CABG. The provided source, however, only discusses therapy after PCI. 2. Different Patient Group: The questions refer to patients generally, specifically those without an indication for oral anticoagulation. The source text focuses on a specific subgroup of patients who *do* have an indication for OAC.

The clinical implications of this failure mode are particularly profound because the system initially appears trustworthy, creating a risk of automation bias. A clinician could receive several correct, guideline-based answers from the RAG tool at the beginning of an encounter, thereby establishing a strong sense of trust. Relying on this initial validation, they might later accept a subtly incorrect—but still confidently presented—answer to the same query without the same level of validation. This pattern, where accuracy suddenly drops without warning, could lead to serious mistakes in medication dosing or treatment plan adjustments, directly risking patient safety. Such unpredictable performance erosion underscores that single-turn accuracy is an insufficient and potentially misleading benchmark for tools intended for conversational clinical use.

To mitigate the risks highlighted by our findings, the architectural design of clinical RAG systems must incorporate more robust safeguards. Based on our analysis, we recommend a multi-layered approach. First, to directly combat the ‘contextual drift’ that caused performance degradation, the dialogue context and the retrieved grounding documents should be stored and versioned independently. This architectural separation would allow the retriever to focus solely on the user’s most recent query for fetching relevant information, preventing the conversational history from contaminating the evidence base. Second, an automated check that validates each generated output against the authoritative source documents should be integrated as a real-time safeguard. This ensures that the system’s outputs remain aligned with the ground truth, regardless of conversational length. Finally, integrating a supplemental fact-verification layer is essential to cross-validate key clinical facts—such as medication dosages or recommendation classes—against a structured, authoritative database. While this study has limitations, including the use of a single RAG architecture, a simulated dialogue, and the use of an automated LLM as the primary evaluator, it highlights the urgent need for more robust validation protocols that account for this contextual dependency.

In conclusion, while RAG systems hold immense promise for delivering evidence-based information in medicine, they are not a panacea for the challenge of AI truthfulness. Our findings serve as a critical cautionary tale, emphasising the necessity of a new standard of reliability, validated through rigorous conversational testing, before these powerful tools can be safely integrated into patient care^13^.

## Methods

### Knowledge source

To evaluate the system’s performance on a clinically relevant and high-stakes task, we used the 2024 ESC Guidelines for the management of chronic coronary syndromes^14^, published by the European Society of Cardiology (ESC) in the European Heart Journal, as the sole external knowledge source for the RAG system.

This document was selected for several key reasons. First, it represents an authoritative, evidence-based, and up-to-date repository of clinical knowledge that is widely used in daily practice by cardiologists and other physicians worldwide. Second, it contains precise, structured, and unambiguous information, particularly regarding complex medication dosing regimens, contraindications, and treatment algorithms. This specificity makes it an ideal ‘ground truth’ for assessing the factual accuracy of an AI-generated response.

### RAG system and experimental design

The architecture of our conversational RAG system and the experimental workflow are depicted in Fig. 1. The system was grounded in the 2024 ESC guideline on chronic coronary syndromes, which was processed for retrieval by segmenting the document into overlapping text chunks (chunk size: 1000 characters, overlap: 200 characters). These chunks were then vectorized using OpenAI’s embedding model (text-embedding-ada-002) and stored in a vector database (Fig. 1A). For the generation step, we utilised the gpt-5-mini model. In line with best practices for RAG systems, which prioritise factuality and reproducibility, the temperature parameter was set to 0 to produce deterministic outputs. When a query was posed, the system retrieved the top-5 most relevant document chunks (*k* = 5) to serve as the evidence base for the generator (Fig. 1C, D).

Our experimental design aimed to assess how accumulating conversational context affects response factuality. First, to simulate a realistic clinical dialogue, a base history of 50 distinct but topically related questions was generated using the OpenAI o3 model and answered by the RAG system. The core experiment then involved posing a single, standardised, multi-part clinical query concerning post-operative antithrombotic therapy after Coronary Artery Bypass Grafting (CABG) (Fig. 1B). This standardised query was tested with progressively longer segments of the pre-existing chat history (e.g., after 10, 20, and up to 50 preceding Q&A pairs), with the chat history influencing both retrieval and generation (Fig. 1C, D). Finally, each response to the standardised query was assessed by an automated evaluator, powered by a more advanced LLM (GPT-5), to determine if it was factually supported by the retrieved source documents (Fig. 1E).

### Outcome and evaluation

The primary outcome was the factual consistency of the RAG system’s response under a fixed conversational context. For each predefined length of dialogue history (0, 10, 20, 30, 40, and 50 Q&A pairs), the identical standardised clinical query was submitted to the RAG system 100 times. Each submission constituted an independent trial to test the stability of the retrieval and generation process. For each of the 100 trials, an automated evaluator, powered by a more advanced LLM (GPT-5), determined if the output was ‘Factual’ or a ‘Hallucination’ based on the retrieved source chunks. The hallucination rate for a given history length was then calculated as the number of ‘Hallucination’ responses out of the 100 trials.

### Ethics approval and consent to participate

Not applicable. This study was a computational experiment using publicly available guidelines (2024 ESC Guidelines) and did not involve human participants or patient data. Therefore, an Internal Review Board (IRB) approval was not required.

## Data Availability

The knowledge source used in this study, the 2024 ESC Guidelines for the management of chronic coronary syndromes, is publicly available. It can be accessed directly at: https://www.escardio.org/Guidelines/Clinical-Practice-Guidelines/Chronic-Coronary-Syndromes#.

## Code availability

The code used for the analyses in this study is available from the corresponding author on reasonable request.
